# Supplementary figures and images for: tRNA sequences can assemble into a replicator
Source: eLife. 2021 Mar 2;10:e63431. doi: 10.7554/eLife.63431 (PMC7924937; doi:10.7554/eLife.63431)

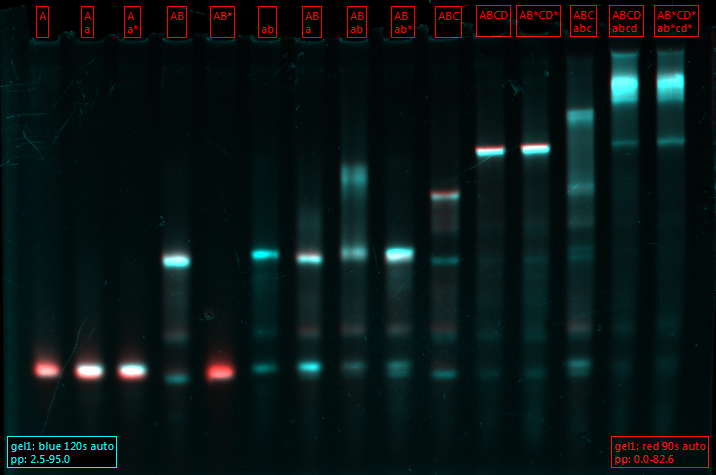

Supplement: Figure 2—source data 1. [file elife-63431-fig2-data1.zip › Figure 2/Fig2_UHU_m562_Annealing_on_5percent_gel_red_cyan.png]

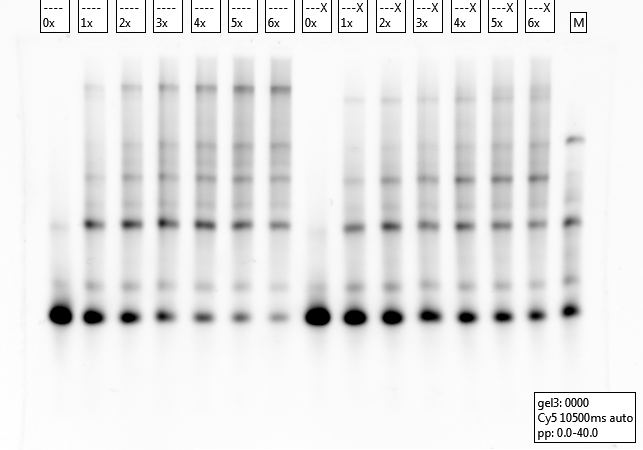

Supplement: Figure 6—source data 1. [file elife-63431-fig6-data1.zip › Figure 6/gel3_Cy5_10500ms_avg1-4_outliers-5-400-sel.roi_rotated_autoint_pp0.0-40.0.tiff]

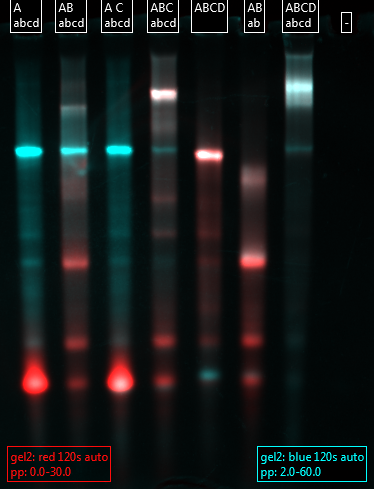

Supplement: Figure 6—figure supplement 1—source data 1. [file elife-63431-fig6-figsupp1-data1.zip › Figure 6-sup1/Fig6-s1_UHU_m562_gel2_red+blue.png]
