# Supplementary material for: tRNA sequences can assemble into a replicator
Source: eLife. 2021 Mar 2;10:e63431. doi: 10.7554/eLife.63431 (PMC7924937; doi:10.7554/eLife.63431)
Supplement: Supplementary file 1. — Strand 0A is 5’-labeled with Cy5, all other strands have a 5’-terminal phosphate. Solid underlines highlight hairpin loops, information domains are indicated by dashed underlines. [file elife-63431-supp1.docx]

**Supplementary file 1. Sequences of all DNA strands used.** *Strand* $0_{A}$ *is 5’-labeled with Cy5, all other strands have a 5’-terminal phosphate. Solid underlines highlight hairpin loops, information domains are indicated by dashed underlines.*

| **Name** | **Sequence (5’ to 3’)** |
| --- | --- |
| $0_{A}$ | GCA G CG TTAATTCCCG CGCCTAT CGGGAATGTAACGC AGTGGGTAATAATGA CGATAGCCGTTCG GGAAAAG CGAACGGT ATCG |
| $1_{A}$ | GCA G CG TTAATTCCCG CGCCTAT CGGGAATGTAACGC AAAAGAAGAGAAAGA CGATAGCCGTTCG GGAAAAG CGAACGGT ATCG |
| $0_{B}$ | GCA G CGAT ACCGTTCG CTTTTCC CGAACGGCTATCGC AGTGGGTAATAATGA GCG A ACTGTCG GTGCTTG CGACAGT GTCGC |
| $1_{B}$ | GCA G CGAT ACCGTTCG CTTTTCC CGAACGGCTATCGC AAAAGAAGAGAAAGA GCG A ACTGTCG GTGCTTG CGACAGT GTCGC |
| $0_{C}$ | GCA G GCGAC ACTGTCG CAAGCAC CGACAGT T CGCC AGTGGGTAATAATGA GCGG TTCCTTGC GGAGTAG GCAAGGAATCCGC |
| $1_{C}$ | GCA G GCGAC ACTGTCG CAAGCAC CGACAGT T CGCC AAAAGAAGAGAAAGA GCGG TTCCTTGC GGAGTAG GCAAGGAATCCGC |
| $0_{D}$ | GCA G GCGGATTCCTTGC CTACTCC GCAAGGAATC GCC AGTGGGTAATAATGA CGTTACATTCCCG ATAGGCG CGGGAATTAA CG |
| $1_{D}$ | GCA G GCGGATTCCTTGC CTACTCC GCAAGGAATC GCC AAAAGAAGAGAAAGA CGTTACATTCCCG ATAGGCG CGGGAATTAA CG |
| ${\overline{\text{0}}}_{\text{A}}$ | GCT G CGC ATTAACGCG CTTGTCC CGCGTTAATTGCGC TCATTATTACCCACT CGCT CTCGGCTG TTTTGCC CAGCCGAGCAGCG |
| ${\overline{\text{1}}}_{\text{A}}$ | GCT G CGC ATTAACGCG CTTGTCC CGCGTTAATTGCGC TCTTTCTCTTCTTTT CGCT CTCGGCTG TTTTGCC CAGCCGAGCAGCG |
| ${\overline{\text{0}}}_{\text{B}}$ | GCT G CGTT GCATTGGC GATCAAA GCCAATGCGAACGC TCATTATTACCCACT CGCAATTAACGCG GGACAAG CGCGTTAAT GCG |
| ${\overline{\text{1}}}_{\text{B}}$ | GCT G CGTT GCATTGGC GATCAAA GCCAATGCGAACGC TCTTTCTCTTCTTTT CGCAATTAACGCG GGACAAG CGCGTTAAT GCG |
| ${\overline{\text{0}}}_{\text{C}}$ | GCT G GTTGGAGAAGGCG AACAGCA CGCCTTC CCAACC TCATTATTACCCACT CGTTCGCATTGGC TTTGATC GCCAATGCAA CG |
| ${\overline{\text{1}}}_{\text{C}}$ | GCT G GTTGGAGAAGGCG AACAGCA CGCCTTC CCAACC TCTTTCTCTTCTTTT CGTTCGCATTGGC TTTGATC GCCAATGCAA CG |
| ${\overline{\text{0}}}_{\text{D}}$ | GCT G CGCTGCTCGGCTG GGCAAAA CAGCCGAG AGCGC TCATTATTACCCACT GTTGG GAAGGCG TGCTGTT CGCCTTCTCCAAC |
| ${\overline{\text{1}}}_{\text{D}}$ | GCT G CGCTGCTCGGCTG GGCAAAA CAGCCGAG AGCGC TCTTTCTCTTCTTTT GTTGG GAAGGCG TGCTGTT CGCCTTCTCCAAC |
